# Morphometric study of the ventricular indexes in healthy ovine BRAIN using MRI

**DOI:** 10.1186/s12917-022-03180-0

**Published:** 2022-03-11

**Authors:** Marco Trovatelli, Carlotta Spediacci, Antonella Castellano, Andrea Bernardini, Daniele Dini, Luca Malfassi, Valentina Pieri, Andrea Falini, Giuliano Ravasio, Marco Riva, Lorenzo Bello, Stefano Brizzola, Davide Danilo Zani

**Affiliations:** 1 Department of Veterinary Medicine and Animal Science (DIVAS), Università degli Studi di Milano, via dell’Università 6, Lodi, Italy; 2 Neuroradiology Unit and CERMAC, Vita-Salute San Raffaele University, IRCCS San Raffaele Scientific Institute, Milan, Italy; 3 Department of Mechanical Engineering, Imperial College London, London, UK; 4 Fondazione La Cittadina Studi e Ricerche Veterinarie, Romanengo, Italy; 5 Department of Medical Biotechnology and Translational Medicine, Università degli Studi di Milano, Milan, Italy; 6 Department of Oncology and Hemato – Oncology, Università degli Studi di Milano, Milan, Italy

**Keywords:** Anatomy, Imaging, Sheep, Brain, MRI

## Abstract

**Background:**

Sheep (*Ovis aries*) have been largely used as animal models in a multitude of specialties in biomedical research. The similarity to human brain anatomy in terms of brain size, skull features, and gyrification index, gives to ovine as a large animal model a better translational value than small animal models in neuroscience. Despite this evidence and the availability of advanced imaging techniques, morphometric brain studies are lacking. We herein present the morphometric ovine brain indexes and anatomical measures developed by two observers in a double-blinded study and validated via an intra- and inter-observer analysis.

**Results:**

For this retrospective study, T1-weighted Magnetic Resonance Imaging (MRI) scans were performed at 1.5 T on 15 sheep, under general anaesthesia. The animals were female *Ovis aries,* in the age of 18-24 months. Two observers assessed the scans, twice time each. The statistical analysis of intra-observer and inter-observer agreement was obtained via the Bland-Altman plot and Spearman rank correlation test.

The results are as follows (mean ± Standard deviation):

Indexes: Bifrontal 0,338 ± 0,032 cm; Bicaudate 0,080 ± 0,012 cm; Evans’ 0,218 ± 0,035 cm; Ventricular 0,241 ± 0,039 cm; Huckman 1693 ± 0,174 cm; Cella Media 0,096 ± 0,037 cm; Third ventricle ratio 0,040 ± 0,007 cm.

Anatomical measures: Fourth ventricle length 0,295 ± 0,073 cm; Fourth ventricle width 0,344 ± 0,074 cm; Left lateral ventricle 4175 ± 0,275 cm; Right lateral ventricle 4182 ± 0,269 cm; Frontal horn length 1795 ± 0,303 cm; Interventricular foramen left 1794 ± 0,301 cm; Interventricular foramen right 1,78 ± 0,317 cm.

**Conclusions:**

The present study provides baseline values of linear indexes of the ventricles in the ovine models. The acquisition of these data contributes to filling the knowledge void on important anatomical and morphological features of the sheep brain.

## Background

Sheep have been largely used in preclinical research as large animal models, due to the anatomical and morphological similarities between the ovine and the human brain.

In order to follow the 3R rules [1] and obtain the best translational value, large animal models are better models than small animals in the neuroscientific research. Small animal models, such as mouse, rat, and rabbit, are usually preferred due to the low cost, ease of care, and the possibility of a high work rate. While they are still valuable for addressing basic research questions, the translation of therapeutic approaches from bench to bed side is often inadequate. Thus, there is a growing awareness that therapies should be tested in large-animal models before clinical application [2]. Limitations of the small animal models are related to anatomical features. A smooth and lissencephalic neocortex characterizes murine models. Accordingly, their gyrification index (GI) -defined as the ratio of total neocortical surface area, including sulci, to superficially exposed neocortical surface area- has a rate of 1.00. Rabbit has a lissencephalic brain type in contrast to the gyrencephalic one and it is characterized by a GI of 1.2 [3]. Other animals are characterised by a higher index. Among them the domestic cat has a GI score of 1.5, the domestic dog 1.73 and the pig with 2.16. Humans exhibit a GI of 2.56 that is more than twice the value of the small animal models while sheep shows a GI of 2.29 [4].

Despite the extensive use of ovine in the biomedical research and the availability of advanced diagnostic imaging techniques such as computed tomography (CT) [5], magnetic resonance imaging (MRI) [6] with diffusion tensor imaging studies (DTI) [7], morphometric brain studies are still lacking. Magnetic resonance imaging has been a widely used diagnostic tool in the study of the central nervous system in sheep. Studies concerning encephalic morphology and tissue volumes with specific interest to the encephalic cortex have been developed and deepened [8–10]. Sheep have been mostly studied from a functional point of view, and a cortical map has been created identifying different areas like motor and sensory areas as in humans [11]. These morphofunctional properties were investigated from structural points. A tractography atlas with the principal white matter fibre bundles was built [7, 10, 12].

Morphometric study of the brain ventricles is commonly used in clinics, since it is a valuable radiological tool for the diagnosis and classification of hydrocephalus or monitoring procedures, such as ventricular shunting. Therefore, the morphometric characteristics of the ovine brain could be a useful reference tool for improving the quality of the research in scientific scenarios, according to the fundamental 3R rules for animal model usage.

Considering this relevant contribution to the scientific community, sheep still lack morphometric data and a comprehensive description of their brain size indexes, thus missing relevant parameters for anatomical arguments. Thorough knowledge of these morphometric features is essential to create an anatomical database for planning research based on sheep as experimental models.

This study aims to establish normal values for bifrontal, bicaudate, ventricular, Huckman’s, Evans’s, third ventricular and cella media index in sheep. Anatomical measures of fourth ventricle’s length and width, left and right lateral ventricle, frontal horn length, left and right Interventricular foramen have also been assessed.

## Results

The results of each value are reported in Table 1.

**Table 1:** *Ventricular indexes of the healthy adult ovine. The main brain indexes are reported with mean and standard deviation*

Ventricular index	Mean	Standard Deviation
Bifrontal	0,338	±0,032
Bicaudate	0,080	±0,012
Evans	0,218	±0,035
Ventricular	0,241	±0,039
Huckman	1693	±0,174
Cella Media	0,096	±0,037
Third ventricle ratio	0,040	±0,007

The value of Bifrontal index is 0,338 ± 0,032 cm. The value is calculated as the maximum distance between anterior horns divided by the maximum internal diameter of frontal bone.

The Bicaudate index is 0,080 ± 0,012 cm. This index is calculated as the minimum bicaudate nuclei distance divided by the maximum distance between anterior horns.

Evans’ index is 0,218 ± 0,035 cm. This index is calculated as the maximum distance between anterior horns divided by the maximum internal skull diameter.

Ventricular index value is 0,241 ± 0,035 cm. This value is the sum of minimum width of the lateral ventricle and the maximum bifrontal diameter.

Huckman index results in 1693 ± 0,174 cm. This value is a measure related to the diameter of anterior ventricular horn. This index is calculated by adding the maximum distance between anterior horns and the minimum bicaudate nuclei distance.

Cella media results in 0,096 ± 0,037 cm. This value is calculated as the ratio of the width of cella media and the maximum inner skull diameter.

Third ventricle index shows is 0,040 ± 0,007 cm. This ratio is calculated between the third ventricle lateral margin and the transverse diameter of the brain, measured along the same line.

In addition to the indexes reported as different ratios, Table 2 reports the results as anatomical measures.

**Table 2:** *Ventricular measures of healthy adult ovine, reported with mean and standard deviation*

Ventricular measure	Mean	Standard Deviation
Fourth ventricle length	0,295	±0,073
Fourth ventricle width	0,344	±0,074
Left lateral ventricle	4175	±0,275
Right lateral ventricle	4182	±0,269
Frontal horn length	1795	±0,303
Interventricular foramen left	1794	±0,301
Interventricular foramen right	1780	±0,317

Tables with the resuming values of the Bland-Altman plots (average bias and limits of agreement) and the Spearman’s rank correlation results (ρ and corresponding p-value) are herein presented. Here, the ‘bias’ is the difference between each pair of measurements, while the ‘limits of agreement’ define the acceptable interval around the average bias. In all Bland-Altman plots, for both the inter and intra-observer variability investigations, more than 95% of the data points lie within the calculated limits of agreement. The complete set of the Bland-Altman are provided in the Supplementary Information.

Inter-observer variability comparing Bland-Altman plot between A_1 time and B_1 time shows perfect agreement between the two observers (Table 3). A clinically small bias, always close to 0, indicates that there is strong agreement between the measurements recorded by the two observers. In the first repetition, there is a complete agreement over all the quantities measured.

**Table 3:** *Mean bias and the limits of agreement per each Bland-Altman in inter-observer variability time1*

Quantity	Lower Limit Agreement	Bias	Upper Limit Agreement	Spearman Rho	P-Value	Corr (Diff/Mean)	Agreement
Bicaudate	−0,0157	−9,15E− 04	0,0138	0,0964	0,7337	✘	✓
Bifrontal	−0,0287	0,0267	0,0821	−0,0321	0,9132	✘	✓
Cella media	−0,0203	0,012	0,0443	−0,4	0,1408	✘	✓
Evans	− 0,0187	0,0249	0,0685	−0,5036	0,0582	✘	✓
Interventricular foramen right [cm]	−0,8841	− 0,2287	0,4273	− 0,1071	0,7048	✘	✓
Interventricular foramen left [cm]	−0,8065	− 0,2427	0,3212	0,2129	0,4462	✘	✓
Fourth ventricle width [cm]	−0,0766	0,0616	0,1998	−0,2821	0,3074	✘	✓
Fourth ventricle length [cm]	−0,1906	0,0539	0,2984	− 0,0857	0,763	✘	✓
Huckmans	−0,1785	0,139	0,4565	−0,4415	0,0995	✘	✓
Frontal horn length [cm]	−0,8268	− 0,246	0,3348	0,1107	0,6953	✘	✓
Third ventricle ratio	−0,0095	0,0072	0,0119	0,2571	0,3538	✘	✓
Left lateral ventricle [cm]	−0,7137	− 0,1567	0,4003	− 0,371	0,1734	✘	✓
Right lateral ventricle [cm]	−0,5946	−0,09	0,4146	−0,4236	0,1156	✘	✓
Ventricular	−0,0791	−0,0276	0,024	−0,0929	0,7435	✘	✓

In the second repetition, most of the measurements agree between the two observers. Non-agreement is exclusively found among the cella media, left frontal horn, right lateral ventricular and ventricular values (Table 4).

**Table 4:** *Mean bias and the limits of agreement per each Bland-Altman in inter-observer variability time 2*

Quantity	Lower Limit Agreement	Bias	Upper Limit Agreement	Spearman Rho	P-Value	Corr (Diff/Mean)	Agreement
Bicaudate	-0,0157	−6,00E− 04	0,0145	− 0,2768	0,3138	✘	✓
Bifrontal	−0,023	0,0277	0,0783	−0,4	0,14	✘	✓
Cellamedia	−0,0127	0,0292	0,0711	0,6179	0,0163	✘	✓
Evans	−0,062	0,0513	0,1646	0,3321	0,2264	✘	✓
Interventricular foramen right [cm]	−0,9394	-0,47	−6,01E− 04	0,4093	0,1298	✘	✓
Interventricular foramen left [cm]	−0,9745	− 0,4753	0,0239	0,4714	0,0783	✘	✓
Fourth ventricle width [cm]	−0,0931	0,0389	0,1708	0,3807	0,1615	✘	✓
Fourth ventricle length [cm]	−0,1293	0,0203	0,1698	0,1698	0,5452	✘	✓
Huckmans	−0,1078	0,1829	0,4737	0,0536	0,8525	✘	✓
Frontal horn length [cm]	−0,9664	− 0,478	0,0104	0,588	0,0211	✓	✘
Third ventricle ratio	−0,0096	0,0053	0,0202	0,1821	0,5151	✘	✓
Left lateral ventricle [cm]	−0,4017	− 0,0227	0,3563	− 0,4826	0,0685	✘	✓
Right lateral ventricle [cm]	−0,3844	0,0167	0,4178	−0,6434	0,0097	✓	✘
Ventricular	−0,1	− 0,0382	0,0236	− 0,6607	0,009	✓	✘

The intra-observer variability has been checked for observer A in two different time points (Table 5). The data show a strong agreement in all the values except for Bicaudate. Although the average bias of the Bicaudate is significantly small, its Bland-Altman plot shows a slight trend confirmed by the Spearman’s ρ and its *p*-value slightly under the threshold of 0.05.

**Table 5:** *Mean bias and the limits of agreement per each Bland-Altman in intra-observer variability observer A*

Quantity	Lower Limit Agreement	Bias	Upper Limit Agreement	Spearman Rho	P-Value	Corr (Diff/Mean)	Agreement
Bicaudate	−0,0114	4,28E− 04	0,0123	0,525	0,0471	✓	✘
Bifrontal	−0,0342	−0,0048	0,0245	-0,0857	0,763	✘	✓
Cella media	-0,0462	−3,02E−04	0,0456	0,1286	0,6482	✘	✓
Evans	−0,1057	− 0,0123	0,0811	−0,4679	0,0809	✘	✓
Interventricular foramen right [cm]	−0,1913	0,044	2,79E− 01	0,3703	0,1743	✘	✓
Interventricular foramen left [cm]	−0,1195	0,0787	0,2769	0,4525	0,0903	✘	✓
Fourth ventricle width [cm]	−0,0931	0,0257	0,1444	−0,3843	0,1573	✘	✓
Fourth ventricle length [cm]	−0,1336	0,0162	0,166	0,22	0,4307	✘	✓
Huckmans	−0,0828	0,0421	0,1669	−0,1036	0,7144	✘	✓
Frontal horn length [cm]	−0,1195	0,0787	0,2769	0,4525	0,0903	✘	✓
Third ventricle ratio	−0,0154	0,0017	0,0066	0,425	0,1159	✘	✓
Left lateral ventricle [cm]	−0,5988	− 0,1993	0,2002	0,479	0,0708	✘	✓
Right lateral ventricle [cm]	−0,5919	−0,1447	0,3026	0,3107	0,2592	✘	✓
Ventricular	−0,0323	−0,0031	0,0262	0,3929	0,1485	✘	✓

Also the intra-observer variability for observer B has been checked in two different time points. The data shows disagreement for cella media, interventricular right foramen and horn length values (Table 6). Although the average bias is relatively small for all these quantities, small trends in the non-agreeing quantities can be seen in the Bland-Altman plots and confirmed by the Spearman’s rank correlation test.

**Table 6:** *Mean bias and the limits of agreement per each Bland-Altman in intra-observer variability observer B*

Quantity	Lower Limit Agreement	Bias	Upper Limit Agreement	Spearman Rho	P-Value	Corr (Diff/Mean)	Agreement
Bicaudate	−0,0151	7,43E− 04	0,0166	0,0571	0,8425	✘	✘
Bifrontal	−0,0527	−0,0039	0,0449	−0,1214	0,6669	✘	✓
Cella media	−0,0501	1,69E− 02	0,0839	0,8929	0	✓	✘
Evans	−0,0393	0,0142	0,0676	0,4821	0,0711	✘	✓
Interventricular foramen right [cm]	−0,7982	− 0,1973	4,04E− 01	0,7138	0,0028	✓	✘
Interventricular foramen left [cm]	−0,5486	− 0,154	0,2406	0,3643	0,1824	✘	✓
Fourth ventricle width [cm]	−0,108	0,0029	0,1139	0,3393	0,216	✘	✓
Fourth ventricle length [cm]	-0,2097	-0,0175	0,1748	0,395	0,1451	✘	✓
Huckmans	-0,2788	0,086	0,4508	0,42	0,1191	✘	✓
Frontal horn length [cm]	-0,5372	0,1533	0,2306	0,529	0,0426	✓	✘
Third ventricle ratio	-0,0107	−1,89E− 04	0,0103	0,2393	0,3892	✘	✓
Left lateral ventricle [cm]	−0,515	− 0,0653	0,3844	0,449	0,0932	✘	✓
Right lateral ventricle [cm]	−0,5173	− 0,038	0,4413	0,2829	0,3069	✘	✓
Ventricular	−0,1009	−0,0137	0,0736	-0,25	0,3677	✘	✓

Overall, the intra- and inter-observer variability results limited. For all values, the differences in the measurements consistently lies within the limits of agreement. In both the inter- and intra-observer studies, the Spearman’s rank correlation tests conclude that for all values, with the few aforementioned exceptions, no correlation is found between the differences of the paired measures and their mean; no proportionate bias is thus recorded.

These analyses show good measure agreement between the two observers, low inter-observer variability and, within the observers, low intra-observer variability, across multiple measurements at different time points. Therefore, the precision, goodness and reliability of the measured quantities can be cleared as acceptable and consistent.

## Discussion

Due to their particular anatomy and size, sheep (*Ovis aries*) have been largely used as animal models in a multitude of specialities in biomedical research ranging from orthopaedics [13], traumatic brain injury [14] and neurological disorders [15].

In terms of neuroanatomical correspondences, sheep exhibit some overlapping features with the human regarding electroencephalographic elements [16], neuroradiological features [17], neurovascular structures [18]. Skull anatomy is also similar to humans in terms of thickness, porosity and the curvature of the calvarium [19].

Considering these characteristics, cadaveric sheep brains have been used as teaching material for mammalian cerebral anatomy [20] and several neurosurgical techniques have been developed and tested through this neurosurgical animal model [21].

Currently, ovine is a reliable large animal model for the study of different human pathologies with reports of cerebral neurodegeneration, neuroinflammation, and intracellular accumulation of storage bodies containing specific proteins [5]. All these pathologies require neuroimaging techniques, such as MRI and CT, to analyse changing in brain size and the ventricular volumes.

In human medicine, ventricular volume is considered the standard measurement of ventricular size, however, a study by Bourne et al. [22] demonstrated a positive correlation with linear measures. The linear indices reported in the manuscript are used in human medicine for slightly different purposes, for example the bicaudate index has been used in several studies to assess caudate atrophy [23], the third ventricular index is the major indicator of third ventricular dilatation [24].

In research studies, a high-quality imaging study paired with volumetric reconstruction is not always feasible and, when it is, it is very time consuming. Additionally, the availability of MRI scanner for large animals is sometimes limited to the high costs and the high level of expertise needed for the large animal model management such as the procedures for general anaesthesia.

Russel et al. [5] reported the third and lateral ventricle volume in sheep. The use of CT, instead of MRI, could limit the discrimination of soft tissue boundaries, with a resulting lower resolution for the ventricle measurements. The intracranial values reported in the manuscript reveal an intracranial volume of 99.9 ± 1.7 ml and a brain volume of 101.1 ± 1.7 ml. Information on volumetric measures are reported, but no mention of linear measurements or indexes, as in our report, has been previously presented until now.

Our results show a standard deviation of the indexes reported ranging from a minimum of 10% to a maximum of 38%. The Cella media is the ratio of the width of the mean cell to the maximum internal diameter of the skull, shows the greatest variability. In our study this value seems to be related to the greater variability of the external diameter of the lateral ventricular bodies, while the variability of the measurements concerning the maximum internal diameter of the skull are minimal.

Cella media index is used as an index for the diagnosis of hydrocephalus [25]. It is interesting to note from our data that if the Cella media index is evaluated together with other indexes such as Evan index and Third ventricle index, the last two indexes do not show much variability. This fact allows us to consider the differences relative to the mean cell as a purely anatomical interspecific difference with no relation to the pressure variations between the subjects of the study. In fact, in the case of an increase in ventricular pressure linked to an increase in ventricular volume or cerebrovascular abnormalities or central atrophy phenomena [26, 27], there should also be a change in the other indices related to the ventricular system.

In human intersex differences are presented and show a lower ventricular volume in female subjects than in male ones [28]. It would be interesting to assess whether these anatomical differences based on the age and sex of the subject also occur in a similar way in the sheep animal model.

In dogs, skull and brain size and morphology differ between brachycephalic, mesocephalic and dolichocephalic breeds. In brachycephalic dogs, a reduction in skull base growth has been documented, which has a potential influence on brainstem morphology and distances measured in this area as well. In a similar way also in the domestic sheep there might be morphometric differences related to individual breeds. Ovine breed skull morphometric differences are reported in literature showing for example Bardhoka breed is characterized by a skull length dimension greater than Hemshin, Morkaraman and Tuj breeds or smaller than the Xisqueta breed [29]. Although the variability of skull size in the sheep breeds is inferior to that in dogs breeds, the fact that this study is focused on a single breed should be considered as a limit.

In the current study, MRI was selected instead of CT for its better spatial resolution and soft-tissue discrimination of intracranial structures. MRI proved a reliable measure quality in ventricular dilation and, for this reason, this technique is the commonly chosen method for the study of disorders of the cerebro-spinal fluid, such as hydrocephalus, in both human [30] and animals [31].

The authors acknowledge the specificity of this study as related to a particular age group. In fact, the animals involved in this work are all adult sheep of 18-24 months old. However, no indications about changes in the brain indexes in the sheep are available, but it is reasonable to argue that differences related to age groups can be found, as occur in humans and in animals such as cat [32] and dog [33]. Russel et al [5] reported changes in the sheep brain volume as increments between 3 and 5 months. This modification seems to stop at approximately 9 months, when another growth phase begins until plateauing at about 15 months of age.

However, it remains important to be aware, as demonstrated in humans [34], that isolated anatomical variations of the lateral ventricles may also be common in sheep and may not be clinically significant. Interpreting radiologists should be aware of morphological variations in the ventricular system.

Further investigations focused on these volumetric changes over time are needed to further improve the current knowledge on ovine as animal model.

## Conclusions

The present study provides reference values of linear indexes of ventricles in the healthy adult ovine animal model. The acquisition of these data with high-filed MRI contributes to the knowledge on essential anatomical and morphological features of sheep brain. These indexes represent relevant normative values for many research projects, focused on various neurological and psychiatric disorders associated with ventriculomegaly or neurodegeneration. In conclusion, this study introduces a useful tool that can represent a normative reference for neuroscientists who aim at exploiting sheep as preclinical model.

## Methods

### Case selection

This is a retrospective study based on 15 MRI scans of female sheep, pecora bergamasca (*ovis aries),* 80 kg weight, in age of 1-2 years old attending at Veterinary Teaching Hospital, Università degli Studi di Milano. Subjects involved in the study were healthy animals with regular vaccination protocol, haemochromocytometric, biochemical and clinical examination within normal limits. Therefore, MRI scans of animals undergoing neurosurgical procedures were excluded from the study.

### Anaesthesia and imaging

Animals underwent anaesthesia for the imaging studies, being inducted via intravenous administration of Diazepam 0,25 mg/Kg + Ketamine 5 mg/Kg, intubated and then maintained under general anaesthesia with isoflurane 2% and oxygen 2 L/min. They were placed in prone position, with the head located in a be-spoke MRI-compatible headframe (Renishaw®). MR imaging was performed on a 1.5 T clinical scanner (Achieva, Philips Healthcare) in a veterinary imaging facility [Fondazione La Cittadina Studi e Ricerche Veterinarie, Romanengo (CR), Italy]. Two small and 2 medium flex coils fixed over both hemispheres were used. The MRI protocol included a T1-weighted volumetric scan for each animal, acquired by using a three- dimensional fast-field-echo (3D-T1 FFE) sequence with the following parameters: TR/TE 25/5 ms; flip angle 40°; voxel size 0.667 × 0.667 × 1.4 mm; SENSE factor R = 2; 150 slices; acquisition time 8 min 40 s.

All the animals emerged from anaesthesia under veterinary care.

### Dataset preparation

Two observers were recruited for this study. Observer A was a PhD in veterinary science with a veterinary surgery background and Observer B was a PhD student in veterinary science with veterinary radiology background. The observers were double-blinded, and all of the measurements were taken independently by the two, in order to study inter-observer variability. Additionally, the two observers repeated the measurements twice, in order to monitor intra-observer variability as well.

**Fig. 1:**
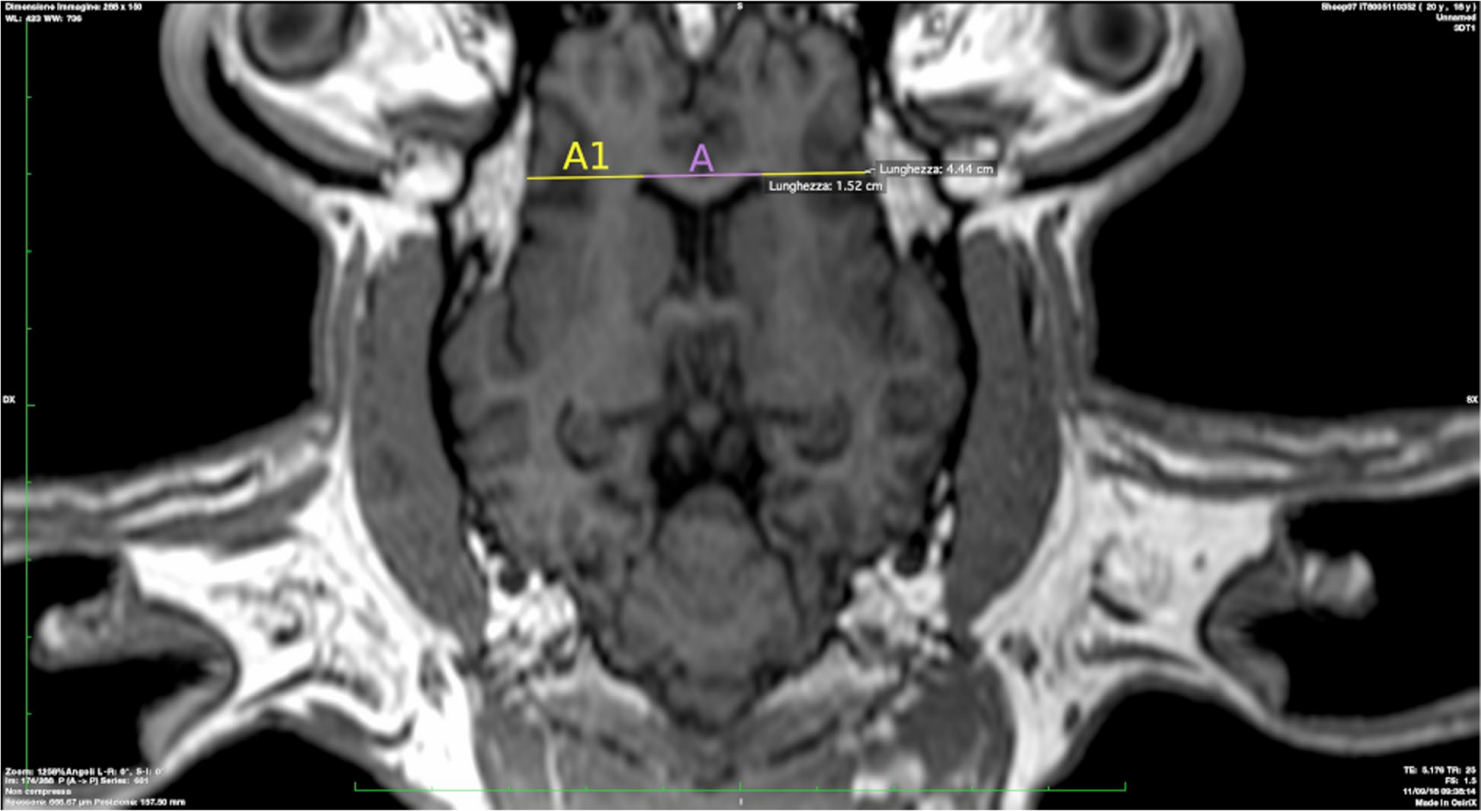
Maximum bifrontal diameter is presented in yellow color (A1). Brain width is presented in purple (A)

Following the work published by Kolsur et al. [35], the acquired images have been analysed to obtain different measures explained below. The indexes calculated was Bifrontal index, Bicaudate index, Evans’ index, Ventricular index, Huckman’s index, Cella media index, Third ventricle Ratio/index. The indexes were then calculated from values:

A: Maximum bifrontal diameter (Fig. 1).

A1: Brain width (Fig. 1).

**Fig. 2:**
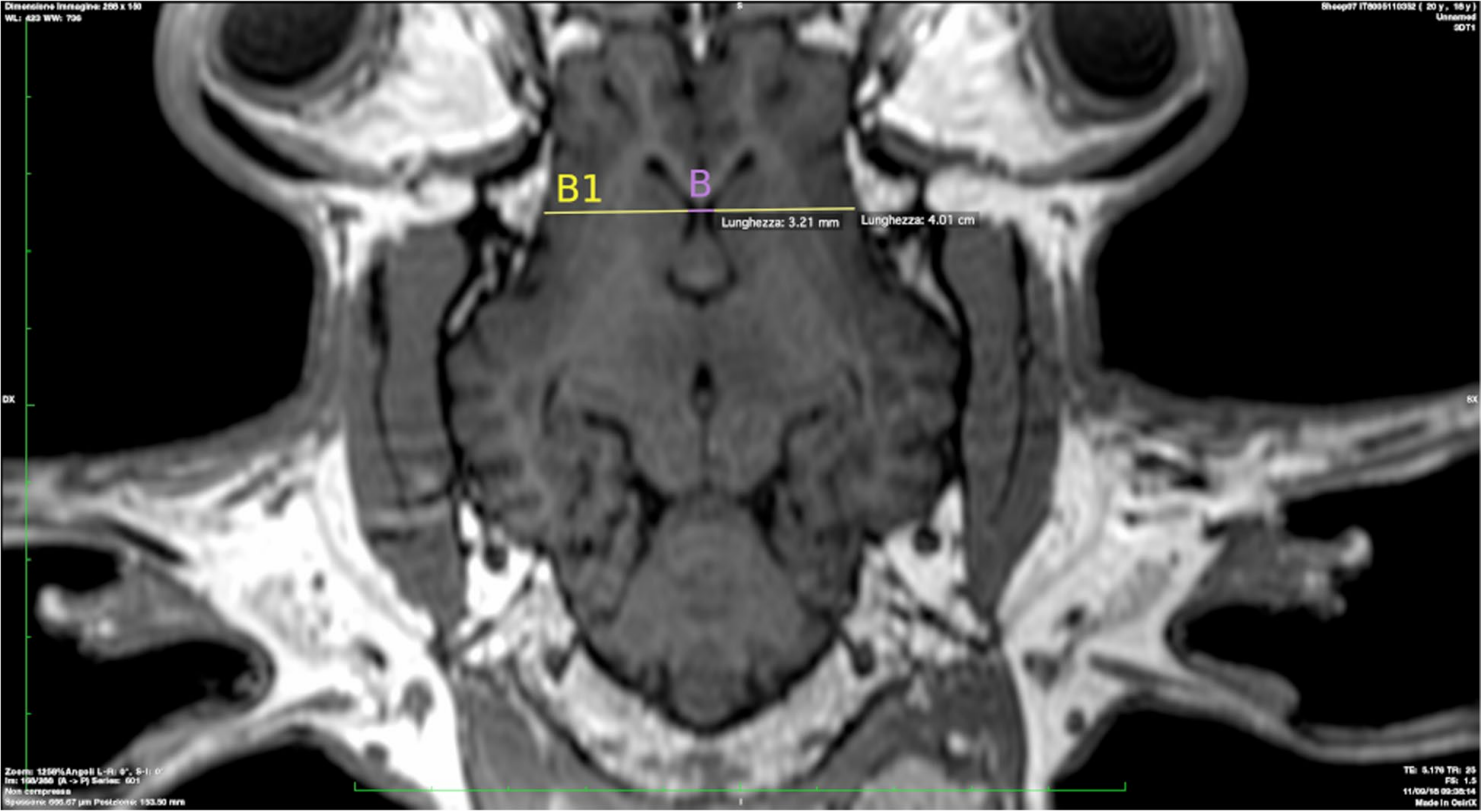
Lateral ventricles minimum width in purple color (B). Brain width is presented in yellow (B1)

B: Lateral ventricles minimum width (Fig. 2).

B1: Brain width (Fig. 2).

**Fig. 3:**
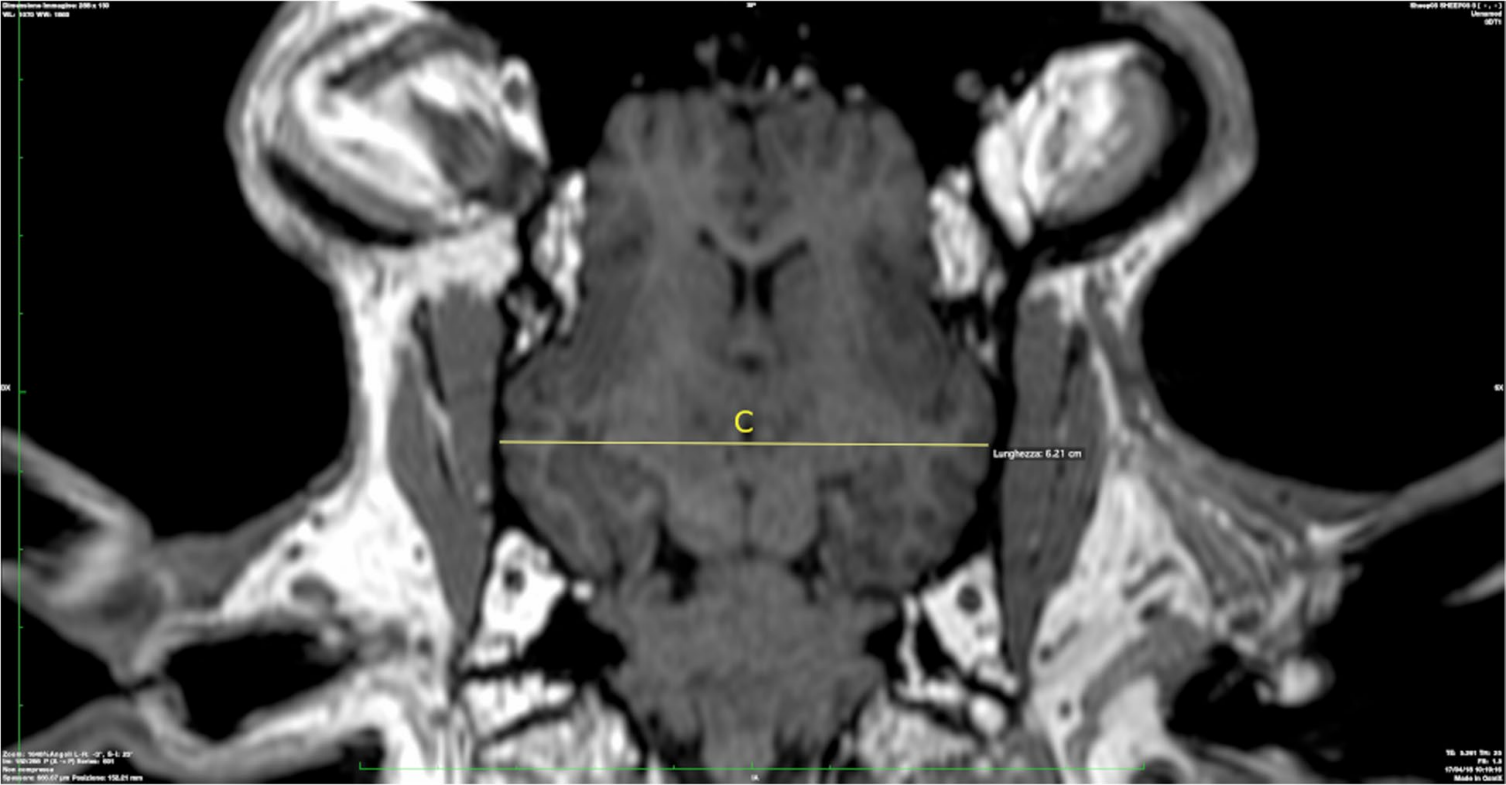
Maximum inner skull diameter

C: Maximum inner skull diameter (Fig. 3).

**Fig. 4:**
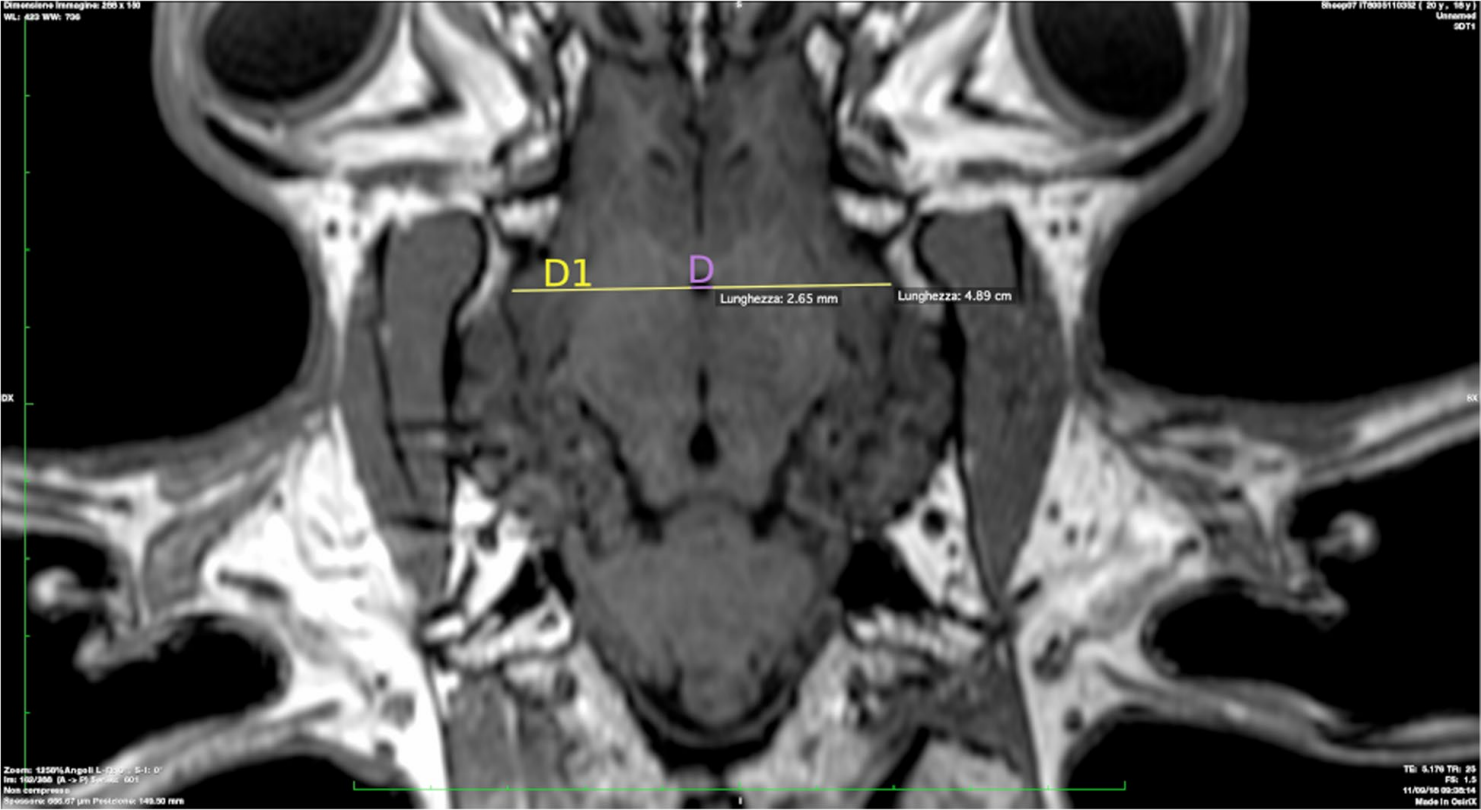
Maximum distances between third ventricle lateral margin is presented in purple color (D). Brain width measured along line D is presented in yellow (D1)

D: Maximum distances between third ventricle lateral margin (Fig. 4).

D1: Brain width measured along line D (Fig. 4).

**Fig. 5:**
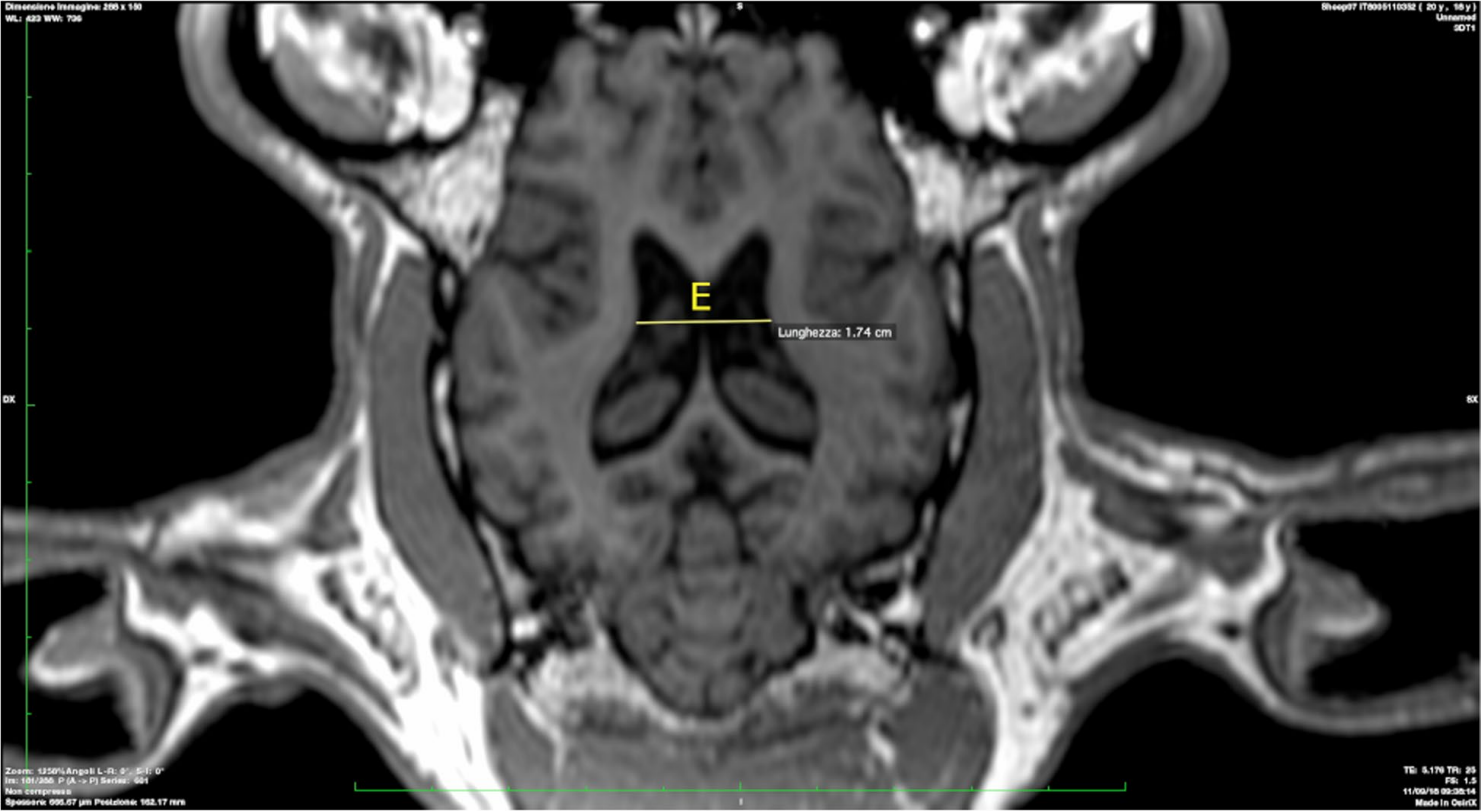
Width of both cella media

E: Width of both cella media (Fig. 5).

### Statistical analyses

**Fig. 6:**
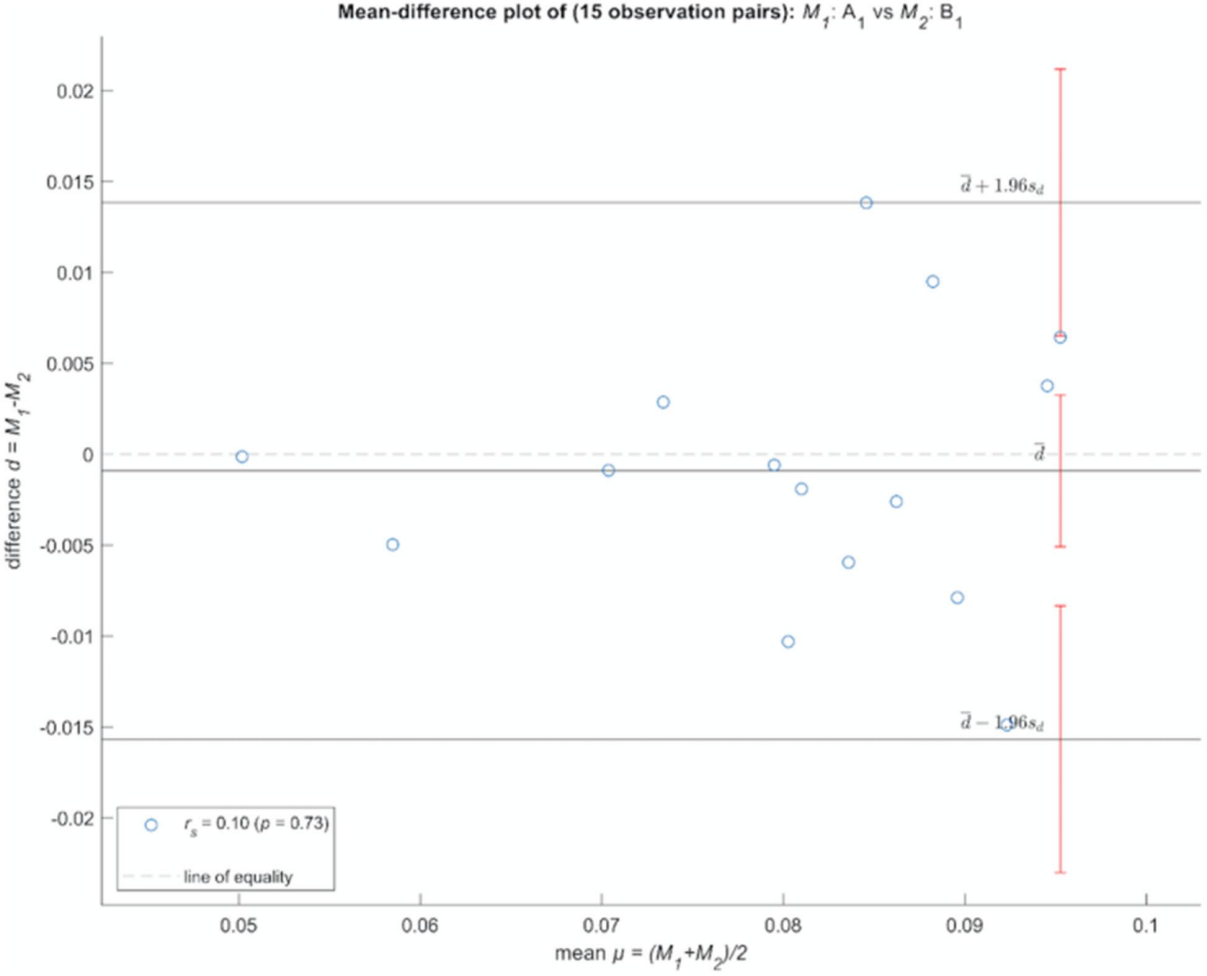
Example of the Bicaudate3 Bland-Altman plot

Statistical analyses were performed using Matlab Software (The Mathworks, I. MATLAB and Statistics Toolbox Release 2018b. (2018)). Firstly, descriptive statistics like mean and standard deviations have been calculated. Then, differences in measurements between the two observers (inter-observer variability), and the differences from the same observer in different time points (intra-observer variability) were compared via Bland-Altman plots [36, 37] (Fig. 6). This allowed to check for any discrepancy in the measurements caused by a poor quality of the measurements or by an inadequate imaging procedure that would hinder the creation of a consistent and reliable dataset of these specific anatomical features.

Here, the average bias (average of the differences between each pair of measurements) has been plotted against the mean of the two measurements. Upper and lower limits of agreement are defined around the bias characterised by an interval of 1.96 times the standard deviation of the bias. At least 95% of the data points should lie within these limits. In addition to each Bland-Altman plot, a spearman rank correlation test was performed to check whether there was a relationship between the bias and the mean. When Spearman’s ρ approaches 0 with values of *p-value* > 0.05, it shows that there is not enough evidence to accept the hypothesis of an existing correlation. Therefore, the null hypothesis of no correlation between the mean and the difference of the measurements cannot be rejected. Accordingly, there is no proportionality in the calculated bias, which means that the two measures agree and are equivalent. Finally, when all these conditions are met, then a good agreement between the two measurements is accepted.

## Data Availability

The datasets used and analysed during the current study are available from the corresponding author on reasonable request.

## Abbreviations

CT: Computed Tomography
DTI: Diffusion Tensor Imaging
GI: Gyrification Index
MRI: Magnetic Resonance Imaging
